# Long-term impacts of 48-h water and feed deprivation on blood and performance responses of grazing *Bos indicus* Nellore heifers

**DOI:** 10.1093/tas/txae015

**Published:** 2024-02-01

**Authors:** Mateus J I Abreu, Rodrigo S Marques, Iorrano A Cidrini, Luis H C Batista, Igor M Ferreira, Karla A Oliveira, Vinicius A Cruz, Arnaldo C Limede, Luciana M Sousa, Matheus Q S França, Gustavo H M Bísio, Gustavo R Siqueira, Flávio D Resende

**Affiliations:** Universidade Estadual Paulista “Júlio de Mesquita Filho” (UNESP), São Paulo, Brazil; School of Animal Sciences, Virginia Polytechnic Institute and State University, Blacksburg, VA, 24061, USA; Universidade Estadual Paulista “Júlio de Mesquita Filho” (UNESP), São Paulo, Brazil; Universidade Estadual Paulista “Júlio de Mesquita Filho” (UNESP), São Paulo, Brazil; Universidade Estadual Paulista “Júlio de Mesquita Filho” (UNESP), São Paulo, Brazil; Universidade Estadual Paulista “Júlio de Mesquita Filho” (UNESP), São Paulo, Brazil; School of Animal Sciences, Virginia Polytechnic Institute and State University, Blacksburg, VA, 24061, USA; School of Animal Sciences, Virginia Polytechnic Institute and State University, Blacksburg, VA, 24061, USA; Universidade Estadual Paulista “Júlio de Mesquita Filho” (UNESP), São Paulo, Brazil; Agência Paulista de Tecnologia dos Agronegócios (APTA), São Paulo, Brazil; Agência Paulista de Tecnologia dos Agronegócios (APTA), São Paulo, Brazil; Universidade Estadual Paulista “Júlio de Mesquita Filho” (UNESP), São Paulo, Brazil; Agência Paulista de Tecnologia dos Agronegócios (APTA), São Paulo, Brazil; Universidade Estadual Paulista “Júlio de Mesquita Filho” (UNESP), São Paulo, Brazil; Agência Paulista de Tecnologia dos Agronegócios (APTA), São Paulo, Brazil

**Keywords:** *Bos indicus*, blood metabolites, feed and water deprivation, grazing, Nellore heifers, performance

## Abstract

The study investigated the effects of 48-h water and feed deprivation on blood and the performance of grazing Nellore (*Bos indicus*) heifers. Twenty-four Nellore heifers (initial body weight [BW] = 238 ± 10 kg; age = 16 ± 2 mo), were ranked by initial BW and age and randomly assigned to one of the two treatments: (1) grazing animals with free access to pasture, water, and mineral-mix (CON; *n *= 12), or (2) the same grazing conditions but deprived of pasture, water, and mineral-mix for 48 h (DPR; *n *= 12). The paddocks consisted of *Urochloa brizantha* cv. Marandu, using a continuous and fixed stocking rate. The experiment lasted 225 d, with the first 14 d considered as the adaptation period (days −14 to −1) and the subsequent 211 d as the evaluation period (days 0 to 211). From days 0 to 2, treatments were applied by keeping the DPR heifers in pens and reintegrating them into the experimental area after a 48-h water and feed deprivation. Individual full BW was recorded on days −14, −13, −1, before (day 0) and after (day 2) treatment application, and on days 6, 11, 12, 41, 42, 210, and 211. Blood samples were collected in the morning on days 0, 2, 6, 12, and 211. A treatment effect was detected (*P *< 0.001) for shrink BW from days 0 to 2, which was greater (*P *< 0.001) in DPR vs. CON heifers. Subsequently, DPR animals were lighter (*P <* 0.001) compared with CON heifers by the end of the deprivation period (day 2). From days 4 to 211, DPR was lighter (*P *< 0.001) compared with CON heifers after treatment application and for the entire experimental period. In the first 10 d after treatment application (days 2 to 12), DPR heifers showed a partial compensatory average daily gain (ADG; *P *< 0.001) compared with CON heifers, while no significant differences were observed in ADG between the treatments from days 12 to 42 and 42 to 211 (*P* > 0.420). Overall ADG (days 2 to 211) was greater (*P *< 0.001) for DPR vs. CON heifers. All serum variables, except AST, were higher (*P *< 0.001) in DPR than in CON heifers on day 2 after treatment application. Our study demonstrates that grazing Nellore heifers subjected to 48-h water and feed deprivation experienced significant alterations in their blood metabolites and BW immediately after the stressful event. Although the deprived heifers partially compensated for their BW loss in the early days post-deprivation, they remained 12 kg lighter than the non-deprived animals throughout the production cycle.

## Teaser

Nellore heifers exposed to 48 h of water and feed deprivation showed a partial compensatory increase in body weight, but they remained lighter than non-deprived heifers throughout the production cycle.

## Introduction

Routine management practices in beef cattle operations, such as shipping and receiving at feedlot facilities, may cause the animals to go without food and water for extended periods (Loerch and Fluharty, 1999; Marques et al., 2012, 2019; Batista et al., 2023). Inflammatory responses might be activated as a result, which has been proven to have detrimental effects on the health and performance of the animals (Carroll and Forsberg, 2007; Cooke, 2017).

It has been well-documented that water and feed deprivation is one factor that stimulates an inflammatory response, neuroendocrine activation, mobilization of reserves, and a limiting factor for cattle performance in the initial weeks following the stressful event (Marques et al., 2012, 2019). Additionally, water and feed deprivation may disturb the ruminal environment, causing microbial death (Meiske et al., 1958), and releasing endotoxins, which may trigger an inflammatory reaction and decrease animal performance (Carroll et al., 2009; Cooke, 2017). This disruption in the microbial community has been linked to reduced feed intake and ruminal fermentative activity (Cole and Hutcheson, 1985; Cole et al., 1986).

Despite studies by Marques et al. (2012; 2019) that have evaluated the effects of feed deprivation on physiological and performance variables, research on this stressor event in *Bos Indicus* cattle raised on tropical pastures is limited. Moreover, *B. indicus* cattle are more predisposed to stress events than *Bos taurus* cattle (Cooke, 2014). To further understand the response in these animals, it is necessary to evaluate the long-term effects of water and feed restriction on the performance and physiological responses of *B. Indicus* cattle raised on tropical pastures. Therefore, we hypothesize that prolonged water and feed deprivation (48 h) of *B. indicus* cattle would limit animal performance and induce changes in blood metabolites. This experiment aimed to evaluate the effects of 48 h of water and feed deprivation on blood metabolites and the performance of pasture-raised Nellore heifers.

## Materials and Methods

The study was conducted following the approved care and handling procedures (protocol 0008/2021) by the Ethics Committee for Animal Use of the Department of Decentralization of Development (CEUA DDD) and adhered to the guidelines established by the Guide for the Care and Use of Agricultural Animals in Research and Teaching (FASS, 2010). The experiment was carried out at the Agência Paulista de Tecnologia dos Agronegócios (APTA) in Colina, SP, Brazil, from April to November 2021. By complying with the ethical and scientific standards, we aimed to ensure the welfare and well-being of the animals involved in the study and the reliability and validity of the results obtained.

### Animals, Treatments, and Experimental Area

Twenty-four Nellore heifers (initial body weight [BW] = 238 ± 10 kg; age = 16 ± 2 mo) were ranked by initial BW and age and randomly assigned to one of the two treatments: (1) grazing animals with free access to pasture, water, and mineral mixture (CON; *n* = 12), or (2) the same grazing conditions but deprived of pasture, water, and mineral mixture for 48 h (DPR; *n* = 12).

The study lasted 225 days, with an adaptation period from days −14 to −1. From days 0 to 2, treatments were applied by keeping the DPR heifers in pens and reintegrating them into the experimental area after a 48-h water and feed deprivation. The 48-h water and feed deprivation was chose to mimic the deprivation time experience by beef cattle during transport and processing time in a feedlot system (Marques et al., 2012; Cooke, 2017). The CON heifers, however, were maintained in the pasture with full access to water, feed, and mineral mixture from days 0 to 2. Upon completion of treatment application (day 2), all heifers returned to their original paddock and were subjected to similar nutritional management throughout the study.

From days −14 to 42, the animals were continuously grazed on a 6-ha paddock of *U. brizantha* cv. Marandu, with a forage mass of 6,727 kg of dry matter (DM)/ha (Table 1), while receiving ad libitum mineral mix (Boi Brasil Nutrição Animal, Indaiatuba, Brazil) contained 15% Ca, 8% P, 13.7% NaCl, 0.6% Mg, 1,266 mg/kg of Cu, 91 mg/kg of I, 1,005 mg/kg of Mn, 9.5 mg/kg of Se, 3,033 mg/kg of Zn, and 1,500 mg/kg of Fe. On day 43, heifers were moved to a 32-ha paddock of *U. brizantha* cv. Marandu with a forage mass of 5,560 kg DM/ha, 6% CP, 75% NDF, and 40% ADF (DM basis). In addition, heifers received a protein supplement (37% CP) at a rate of 1g/kg of BW, containing (as-fed basis) 30% of corn, 57% of cottonseed meal, 7% of urea, and 6% of the same mineral mix aforementioned (Table 1). Heifers were maintained in this condition until the end of the trial (day 211).

**Table 1.** Forage mass (*Urochloa brizantha* cv. Marandu) and chemical composition of supplements and pastures during the experimental period (DM basis)

Item	Pasture A^1^	Protein supplement ^2^	Pasture B ^2^
Forrage mass^3^, kg/ha	6727	-	5560
Dry matter, %	28.3	87.4	82.6
Crude protein, %	10	37	6.7
Neutral detergent fiber, %	73	29	75
Acid detergent fiber, %	33	13	40

^1^From day −14 to 42, the animals were continuously grazed on a 6-ha paddock, while receiving ad libitum mineral mix (Boi Brasil Nutricao Animal, Indaiatuba, Brazil) contained 15% Ca, 8% P, 13.7% NaCl, 0.6% Mg, 1,266 mg/kg of Cu, 91 mg/kg of I, 1,005 mg/kg of Mn, 9.5 mg/kg of Se, 3,033 mg/kg of Zn, and 1,500 mg/kg of Fe.

^2^On day 43, heifers were moved to a 32-ha paddock of *Urochloa brizantha* cv. Marandu and received a protein supplement (37% CP) at a rate of 1g/kg of BW, containing (as-fed basis) 30% of corn, 57% of cottonseed meal, 7% of urea, and 6% of the same mineral mix aforementioned.

^3^Forage mass was characterized through a double sampling method as described by Sollenberger and Cherney (1995). Hand-plucked samples were used to estimate the nutritional value of the forage (De Vries, 1995).

### Sampling, Laboratory Analyses, and Measurements

Individual full BW was recorded on days −14, −13, −1, before (day 0) and after (day 2) treatment application, and on days 6, 11, 12, 41, 42, 210, and 211. Individual BW collected on days 0 and 2 were used to evaluate shrink BW associated with treatment application. To calculate the average daily gain (ADG), the difference between the mean weights in each time interval was divided by the corresponding number of days in the intervals between days 2 and 12, 12 and 42, and 42 and 211.

Blood samples were collected in the morning on days 0 (before treatment application), 2 (immediately at the end of treatment application and before full access to feed and water), 6, 12, and 211 via jugular venipuncture into commercial blood collection tubes (Vacuette; Greiner Bio-One, Americana, SP, Brazil). All blood samples were placed immediately on ice, centrifuged (2,800 × *g* for 10 min; 4 °C) for serum harvest, and stored at −80 °C on the same day of collection. Serum concentrations were analyzed for non-esterified fatty acids (NEFA), glucose, urea, albumin, total protein, and aspartate aminotransferase (AST). Serum concentrations of NEFA were determined using a commercial colorimetric kit (HR Series NEFA-2; Wako Pure Chemical Industries Ltd., Richmond, VA, USA) with modifications described by Pescara et al. (2010). Plasma glucose concentrations were obtained as described by Cappellozza et al. (2014). Urea, albumin, and total protein were analyzed using commercial biochemical kits (Bioclin; Quibasa - Química Básica Ltda, Belo Horizonte, MG, Brazil) and a biochemical analyzer (Cobas Mira Plus; Roche Diagnostic Systems), while AST was analyzed with the Bioclin kit (Quibasa - Química Básica Ltda, Belo Horizonte, MG, Brazil).

As all heifers were grazing in the same paddock, the forage mass was characterized through a double sampling method as described by Sollenberger and Cherney (1995). Hand-plucked samples were used to estimate the nutritional value of the forage (De Vries, 1995). These samples were partially dried in a forced-air circulation oven set at 55 °C for 72 h, ground through a Thomas Model 4 Wiley mill (Thomas Scientific, Swedesboro, NJ, USA) to pass through 2- and 1-mm screen sieves, and then stored for chemical analysis. Both the forage and supplement components were analyzed to evaluate the (DM; method 934.01), mineral matter (method 942.05), and (CP; method 978.04), which were measured according to AOAC (1995). Forage NDF was evaluated as described by Robertson and Van Soest (1981), using a Tecnal® TE-149 fiber analyzer (Piracicaba, São Paulo, BRA). Cellulose was solubilized with 72% sulfuric acid, and the lignin was derived from the difference (Goering and Van Soest, 1970).

### Statistical Analysis

For statistical analysis, the mathematical assumptions of data normality (Shapiro–Wilk test) and homogeneity of variance (Bartlett test) were initially verified. All data were analyzed using the animal as the experimental unit and Satterthwaite approximation to determine the denominator df for the tests of fixed effects. Performance and serum variables data were analyzed using the MIXED procedure of SAS (SAS Inst., Inc., Cary, NC, USA). The model statement used for BW, ADG, and serum variables contained the effects of treatment, day, and treatment × day interaction, using heifer(treatment) as the random variable and with day as the specified term for the repeated statement and heifer(treatment) as the subject. The covariance structure used was the first-order autoregressive, which provided the smallest Akaike information criterion and hence the best fit for all variables analyzed. All results are reported as least square means for values and separated using PDIFF. Significance was set at *P* ≤ 0.05, and tendencies were determined if *P* > 0.05 and ≤ 0.10. Results are reported according to the main effects if no interactions were significant.

## Results

**Figure 1.**
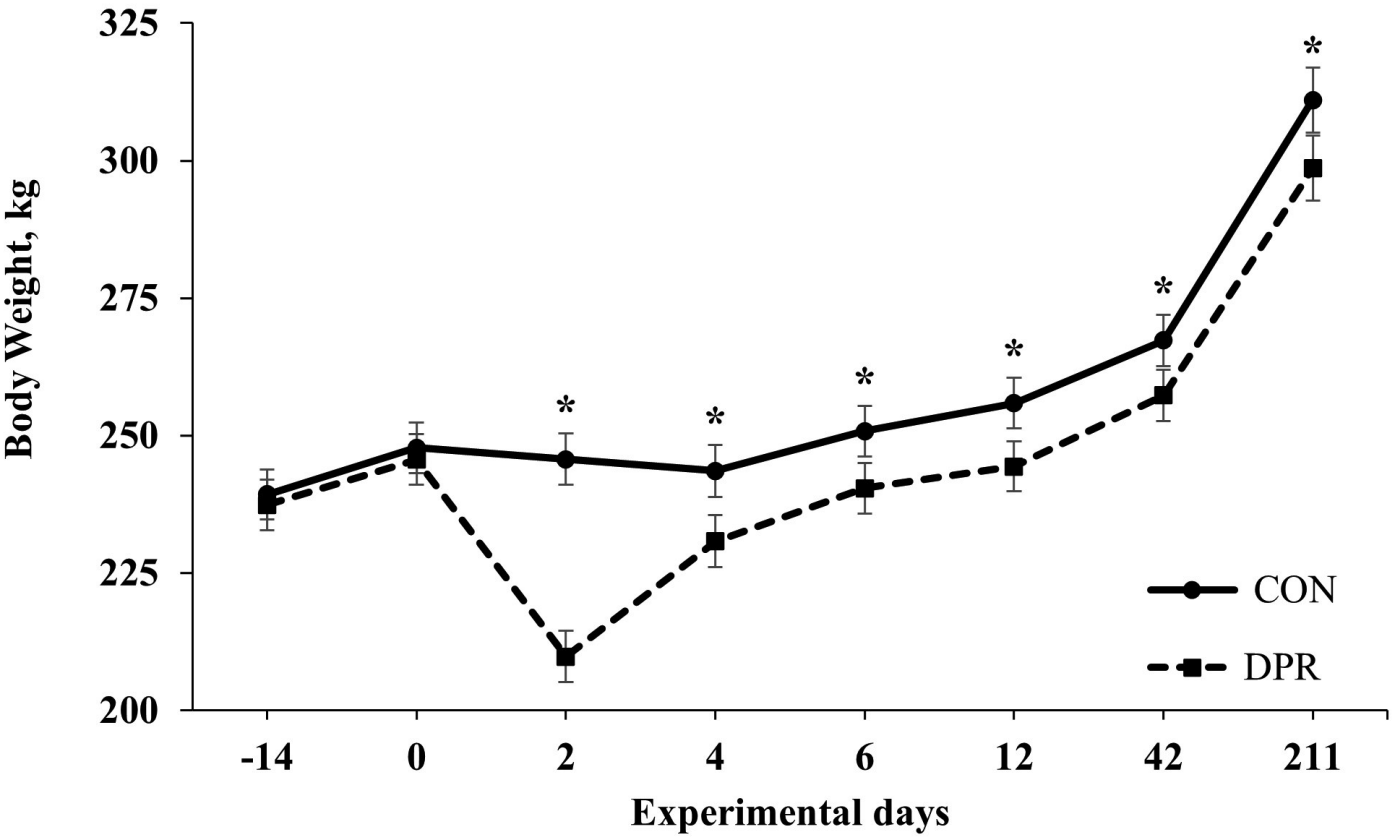
Body weight of grazing Nellore heifers submitted to (A) full access to pasture, water, and mineral mixture (CON; *n* = 12) or (B) maintained under equal grazing conditions whereas deprived of pasture, water, and mineral mixture for 48 h (DPR; *n* = 12). Treatments were applied from days 0 to 2. From days −14 to 42, the animals were continuously grazed on a 6-ha paddock, while receiving ad libitum mineral mix (Boi Brasil Nutricao Animal, Indaiatuba, Brazil) contained 15% Ca, 8% P, 13.7% NaCl, 0.6% Mg, 1,266 mg/kg of Cu, 91 mg/kg of I, 1,005 mg/kg of Mn, 9.5 mg/kg of Se, 3,033 mg/kg of Zn, and 1,500 mg/kg of Fe. On day 43, heifers were moved to a 32-ha paddock of *Urochloa brizantha* cv. Marandu and received a protein supplement (37% CP) at a rate of 1g/kg of BW, containing (as-fed basis) 30% of corn, 57% of cottonseed meal, 7% of urea, and 6% of the same mineral mix aforementioned. Treatment × day interaction (*P* < 0.001) was detected. Within days: *P < 0.05.

**Figure 2.**
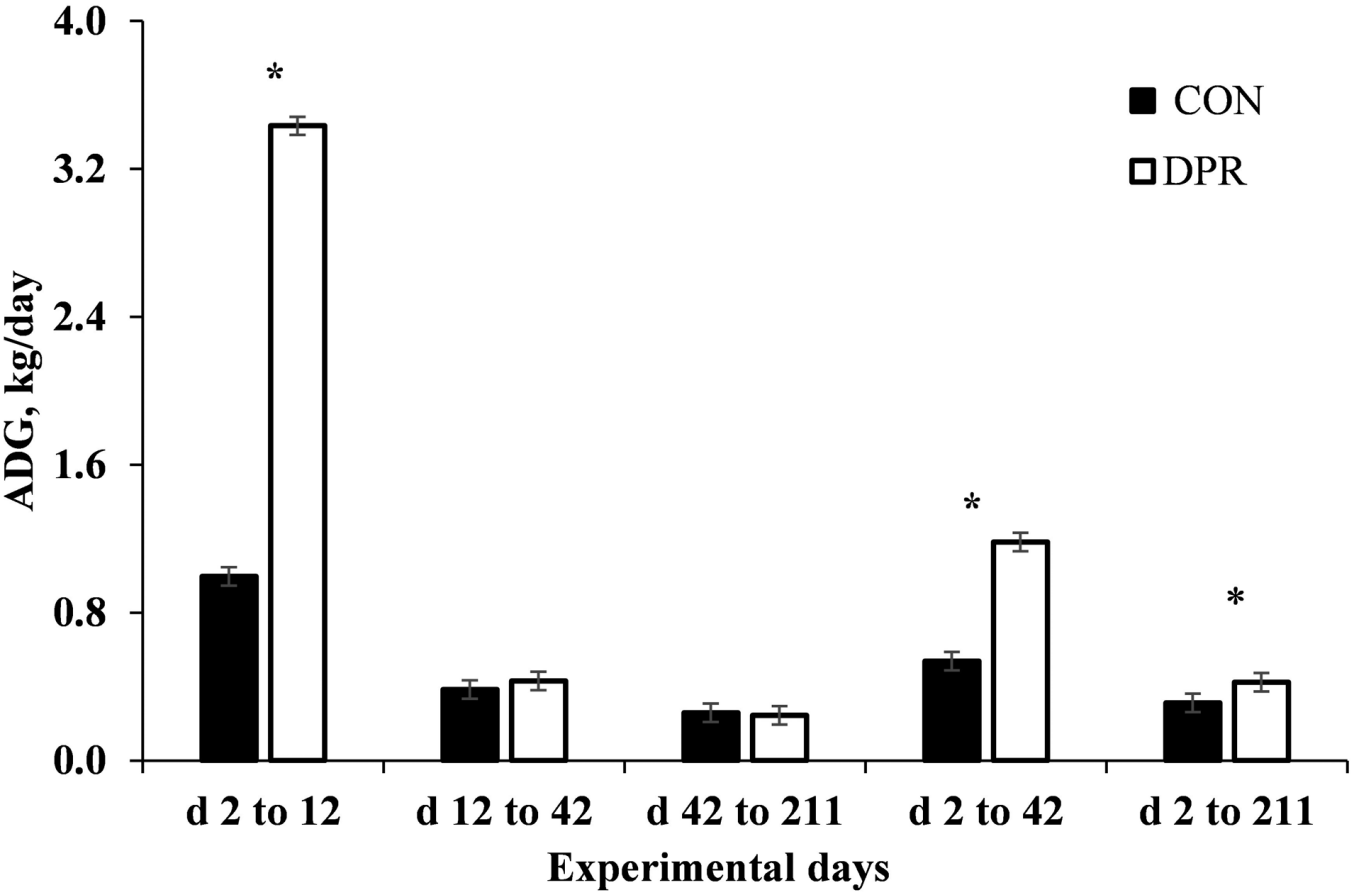
Average daily gain of grazing Nellore heifers submitted to (A) full access to pasture, water, and mineral mixture (CON; *n* = 12) or (B) maintained under equal grazing conditions whereas deprived of pasture, water, and mineral mixture for 48 h (DPR; *n* = 12). Treatments were applied from days 0 to 2. From days −14 to 42, the animals were continuously grazed on a 6-ha paddock, while receiving ad libitum mineral mix (Boi Brasil Nutricao Animal, Indaiatuba, Brazil) contained 15% Ca, 8% P, 13.7% NaCl, 0.6% Mg, 1,266 mg/kg of Cu, 91 mg/kg of I, 1,005 mg/kg of Mn, 9.5 mg/kg of Se, 3,033 mg/kg of Zn, and 1,500 mg/kg of Fe. On day 43, heifers were moved to a 32-ha paddock of *Urochloa brizantha* cv. Marandu and received a protein supplement (37% CP) at a rate of 1g/kg of BW, containing (as-fed basis) 30% of corn, 57% of cottonseed meal, 7% of urea, and 6% of the same mineral mix aforementioned. Treatment × day interaction (*P* < 0.001) was detected. Within days: *P < 0.01.

Initially, BW did not differ (*P* = 0.64) among treatments (Table 2) as designed. A treatment effect was detected (*P *< 0.001) for shrunk BW from days 0 to 2, which was greater (*P *< 0.001) in DPR compared with CON heifers (Table 2). Subsequently, a treatment × day interaction was detected (*P *< 0.001) for BW (Figure 1), where DPR animals were lighter (*P <* 0.001) compared with CON heifers by the end of the deprivation period (day 2) and to the remaining of the experiment. For instance, from days 4 to 21, DPR heifers reduced the difference in BW, whereas DPR was still lighter (*P* < 0.001) compared with CON heifers after treatment application and for the entire experimental period (Table 2; Figure 1). In the first 10 d after treatment application (days 2 to 12), DPR heifers exhibited a partial compensatory ADG (*P* < 0.001) compared with CON heifers, whereas no difference was observed (*P* > 0.296) from days 12 to 42 and 42 to 211 between treatments (Table 1; Figure 2). However, the overall ADG (days 2 to 211) was greater (*P *< 0.001) for DPR compared with CON heifers. Despite this difference in ADG between treatments, DPR heifers were still lighter (*P *< 0.001) than the CON group by the end of the evaluation period (Table 2; Figure 2).

**Table 2.** Performance and blood variables of grazing Nellore heifers submitted to (A) full access to pasture, water, and mineral mixture (CON; *n* = 12) or (B) maintained under equal grazing conditions whereas deprived of pasture, water, and mineral mixture for 48 h (DPR; *n* = 12)^1^

Item	Treatments		SEM	*P*-value		
	CON	DPR		Treatment	Day	Treatment × day
*Performance*						
Body weight, kg						
day −14	239	237	4.58	0.692	—	—
day 0	248	246	4.61	0.642	—	—
day 2	246	210	4.64	<0.001	—	—
day 4	244	231	4.74	0.011	—	—
day 6	251	240	4.56	0.033	—	—
day 12	256	244	4.58	0.018	—	—
day 42	267	257	4.69	0.042	—	—
day 211	311	299	0.54	<0.001	—	—
Average daily gain,^2^ kg/d	0.311	0.424	0.01	<0.001	<0.001	<0.001
Shrunk body weight,^3^ kg	0.85	14.6	0.54	<0.001	—	—
*Blood variables* ^4^						
Albumin, g/dL	3.08	3.14	0.03	0.124	<0.001	0.049
Total protein, g/dL	6.39	6.59	0.11	0.194	<0.001	0.025
NEFA, mmol/L	0.63	0.79	0.03	0.004	<0.001	<0.001
Glucose, mg/dL	90.7	90.7	1.80	0.994	<0.001	0.063
Urea, mg/dL	7.46	13.7	0.31	<0.001	<0.001	<0.001
Aspartate aminotransferase, U/L	72.2	72.3	2.23	0.974	0.030	0.694

^1^Treatments were applied from days 0 to 2. From days −14 to 42, the animals were continuously grazed on a 6-ha paddock, while receiving ad libitum mineral mix (Boi Brasil Nutricao Animal, Indaiatuba, Brazil) contained 15% Ca, 8% P, 13.7% NaCl, 0.6% Mg, 1,266 mg/kg of Cu, 91 mg/kg of I, 1,005 mg/kg of Mn, 9.5 mg/kg of Se, 3,033 mg/kg of Zn, and 1,500 mg/kg of Fe. On day 43, heifers were moved to a 32-ha paddock of *Urochloa brizantha* cv. Marandu and received a protein supplement (37% CP) at a rate of 1g/kg of BW, containing (as-fed basis) 30% of corn, 57% of cottonseed meal, 7% of urea, and 6% of the same mineral mix aforementioned.

^2^Calculated using days 2 and 211.

^3^Based on BW loss from days 0 to 2.

^4^Blood samples were collected in the morning on days 0 (before treatment application), 2 (immediately at the end of treatment application and before full access to feed and water), 6, 12, and 211 via jugular venipuncture into commercial blood collection tubes (Vacuette; Greiner Bio-One, Americana, SP, Brazil). All blood samples were placed immediately on ice, centrifuged (2,800 × *g* for 10 min; 4 °C) for serum harvest, and stored at −80 °C on the same day of collection.

**Figure 3.**
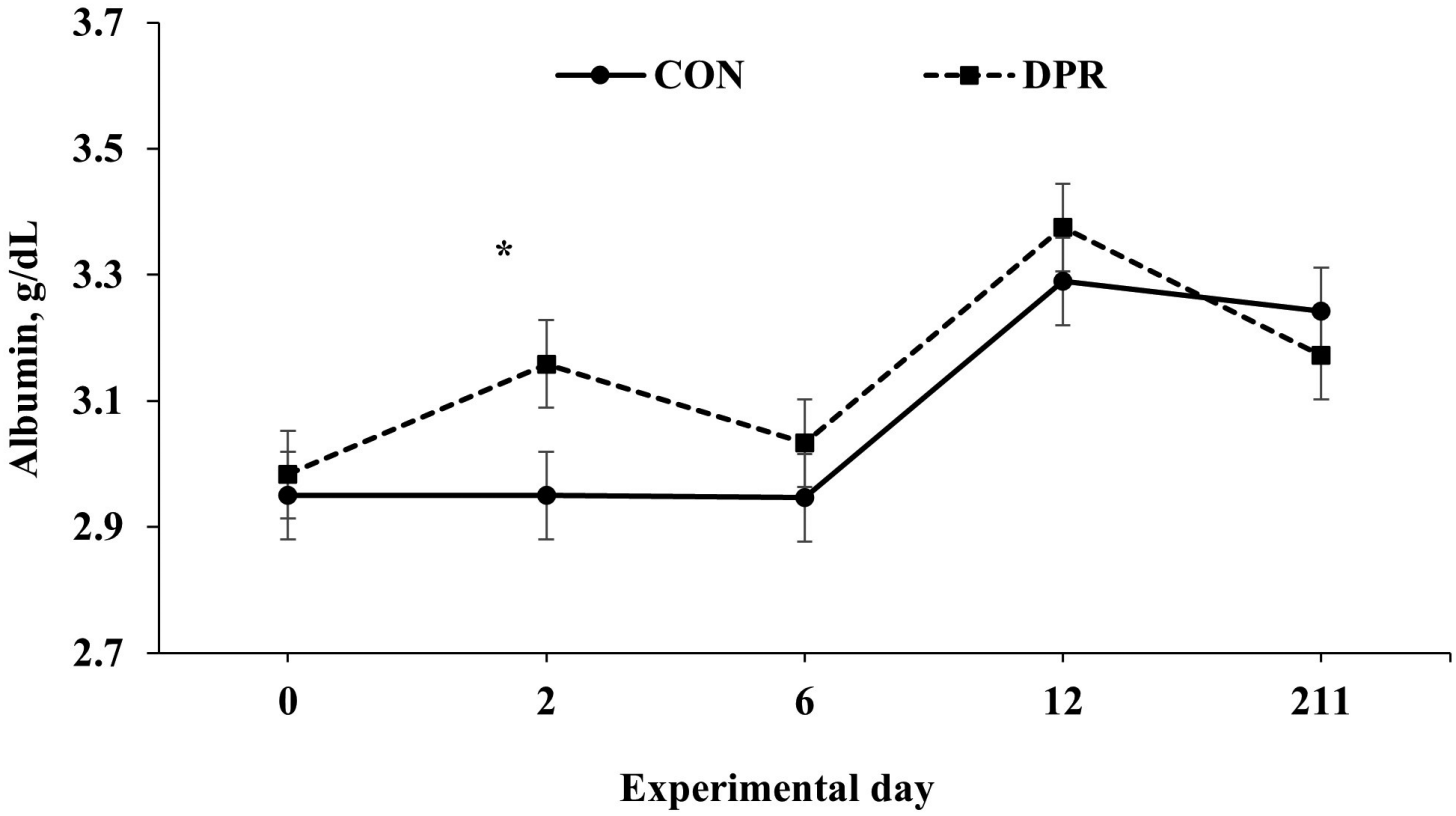
Serum albumin concentration in grazing Nellore heifers submitted to (A) full access to pasture, water, and mineral mixture (CON; *n* = 12) or (B) maintained under equal grazing conditions whereas deprived of pasture, water, and mineral mixture for 48 h (DPR; *n* = 12). Treatments were applied from days 0 to 2. From days −14 to 42, the animals were continuously grazed on a 6-ha paddock, while receiving ad libitum mineral mix (Boi Brasil Nutricao Animal, Indaiatuba, Brazil) contained 15% Ca, 8% P, 13.7% NaCl, 0.6% Mg, 1,266 mg/kg of Cu, 91 mg/kg of I, 1,005 mg/kg of Mn, 9.5 mg/kg of Se, 3,033 mg/kg of Zn, and 1,500 mg/kg of Fe. On day 43, heifers were moved to a 32-ha paddock of *Urochloa brizantha* cv. Marandu and received a protein supplement (37% CP) at a rate of 1g/kg of BW, containing (as-fed basis) 30% of corn, 57% of cottonseed meal, 7% of urea, and 6% of the same mineral mix aforementioned. Treatment × day interaction (*P* < 0.001) was detected. Within days: *P < 0.01.

**Figure 4.**
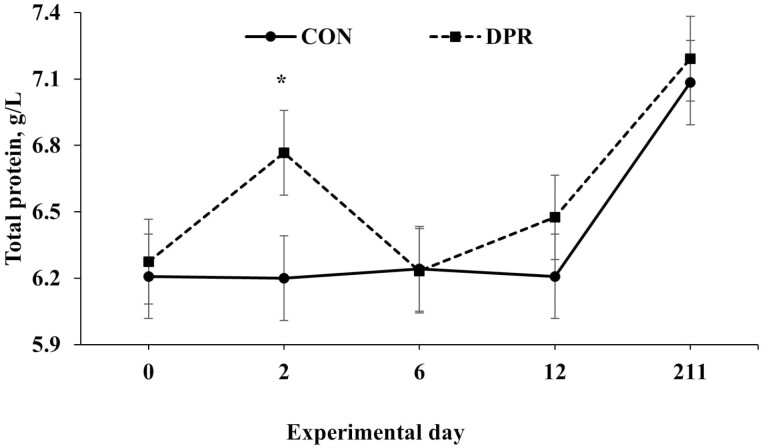
Serum total protein concentration in grazing Nellore heifers submitted to (A) full access to pasture, water, and mineral mixture (CON; *n* = 12) or (B) maintained under equal grazing conditions whereas deprived of pasture, water, and mineral mixture for 48 h (DPR; *n* = 12). Treatments were applied from days 0 to 2. From days −14 to 42, the animals were continuously grazed on a 6-ha paddock, while receiving ad libitum mineral mix (Boi Brasil Nutricao Animal, Indaiatuba, Brazil) contained 15% Ca, 8% P, 13.7% NaCl, 0.6% Mg, 1,266 mg/kg of Cu, 91 mg/kg of I, 1,005 mg/kg of Mn, 9.5 mg/kg of Se, 3,033 mg/kg of Zn, and 1,500 mg/kg of Fe. On day 43, heifers were moved to a 32-ha paddock of *Urochloa brizantha* cv. Marandu and received a protein supplement (37% CP) at a rate of 1g/kg of BW, containing (as-fed basis) 30% of corn, 57% of cottonseed meal, 7% of urea, and 6% of the same mineral mix aforementioned. Treatment × day interaction (*P* < 0.001) was detected. Within days: *P < 0.01.

**Figure 5.**
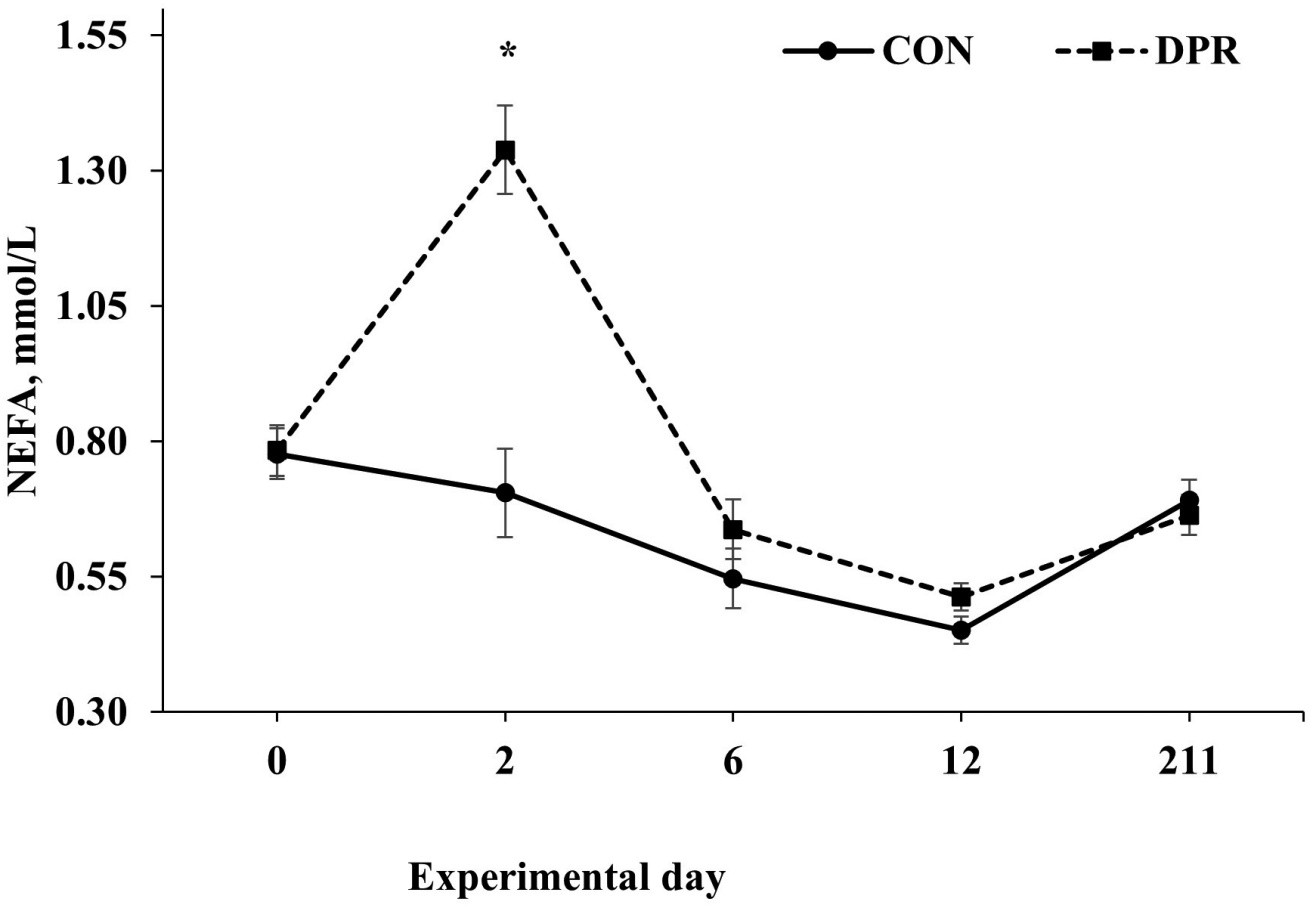
Serum NEFA concentration in grazing Nellore heifers submitted to (A) full access to pasture, water, and mineral mixture (CON; *n* = 12) or (B) maintained under equal grazing conditions whereas deprived of pasture, water, and mineral mixture for 48 h (DPR; *n* = 12). Treatments were applied from days 0 to 2. From days −14 to 42, the animals were continuously grazed on a 6-ha paddock, while receiving ad libitum mineral mix (Boi Brasil Nutricao Animal, Indaiatuba, Brazil) contained 15% Ca, 8% P, 13.7% NaCl, 0.6% Mg, 1,266 mg/kg of Cu, 91 mg/kg of I, 1,005 mg/kg of Mn, 9.5 mg/kg of Se, 3,033 mg/kg of Zn, and 1,500 mg/kg of Fe. On day 43, heifers were moved to a 32-ha paddock of *Urochloa brizantha* cv. Marandu and received a protein supplement (37% CP) at a rate of 1g/kg of BW, containing (as-fed basis) 30% of corn, 57% of cottonseed meal, 7% of urea, and 6% of the same mineral mix aforementioned. Treatment × day interaction (*P* < 0.001) was detected. Within days: *P < 0.01.

**Figure 6.**
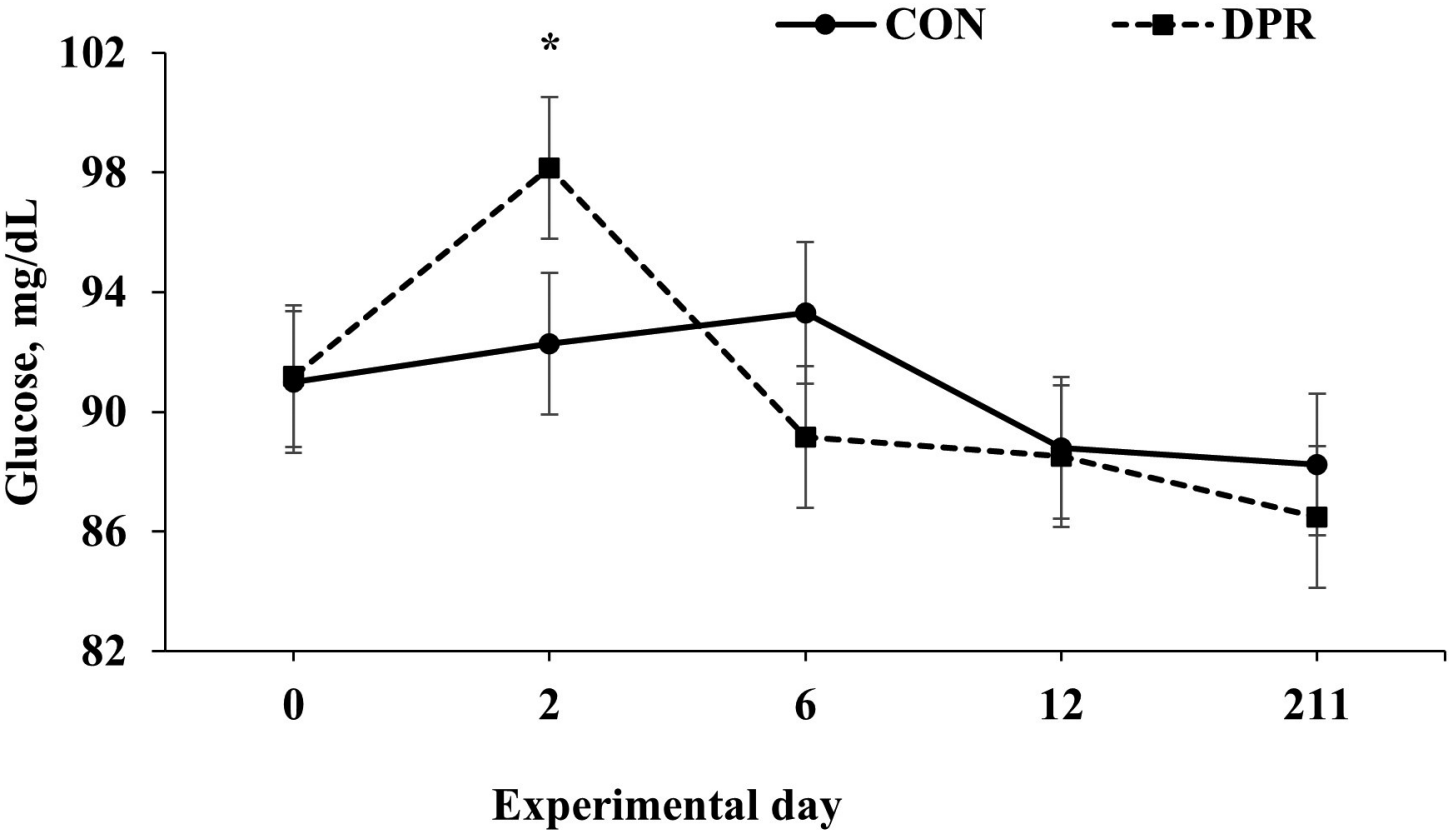
Serum glucose concentration in grazing Nellore heifers submitted to (A) full access to pasture, water, and mineral mixture (CON; *n* = 12) or (B) maintained under equal grazing conditions whereas deprived of pasture, water, and mineral mixture for 48 h (DPR; *n* = 12). Treatments were applied from days 0 to 2. From days −14 to 42, the animals were continuously grazed on a 6-ha paddock, while receiving ad libitum mineral mix (Boi Brasil Nutricao Animal, Indaiatuba, Brazil) contained 15% Ca, 8% P, 13.7% NaCl, 0.6% Mg, 1,266 mg/kg of Cu, 91 mg/kg of I, 1,005 mg/kg of Mn, 9.5 mg/kg of Se, 3,033 mg/kg of Zn, and 1,500 mg/kg of Fe. On day 43, heifers were moved to a 32-ha paddock of *Urochloa brizantha* cv. Marandu and received a protein supplement (37% CP) at a rate of 1g/kg of BW, containing (as-fed basis) 30% of corn, 57% of cottonseed meal, 7% of urea, and 6% of the same mineral mix aforementioned. Tendency for treatment × day interaction (*P* < 0.001) was detected. Within days: * *P* = 0.06.

**Figure 7.**
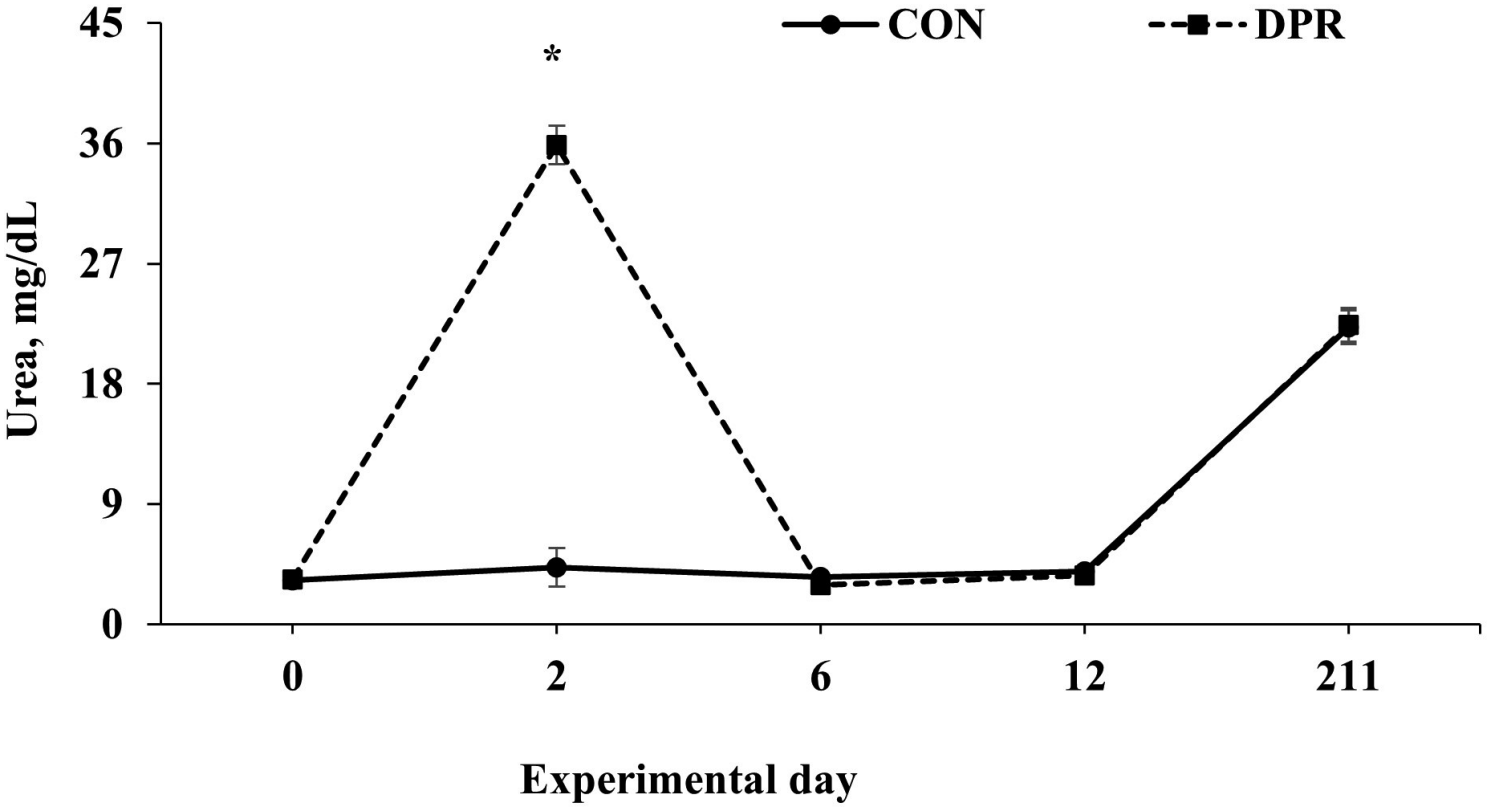
Serum urea concentration in grazing Nellore heifers submitted to (A) full access to pasture, water, and mineral mixture (CON; *n* = 12) or (B) maintained under equal grazing conditions whereas deprived of pasture, water, and mineral mixture for 48 h (DPR; *n* = 12). Treatments were applied from days 0 to 2. From days −14 to 42, the animals were continuously grazed on a 6-ha paddock, while receiving ad libitum mineral mix (Boi Brasil Nutricao Animal, Indaiatuba, Brazil) contained 15% Ca, 8% P, 13.7% NaCl, 0.6% Mg, 1,266 mg/kg of Cu, 91 mg/kg of I, 1,005 mg/kg of Mn, 9.5 mg/kg of Se, 3,033 mg/kg of Zn, and 1,500 mg/kg of Fe. On day 43, heifers were moved to a 32-ha paddock of *Urochloa brizantha* cv. Marandu and received a protein supplement (37% CP) at a rate of 1g/kg of BW, containing (as-fed basis) 30% of corn, 57% of cottonseed meal, 7% of urea, and 6% of the same mineral mix aforementioned. Treatment × day interaction (*P* < 0.001) was detected. Within days: * *P* < 0.01.

Treatment × day interactions (*P* ≤ 0.049) were found for several serum variables, including albumin, total protein, NEFA, and urea, while glucose showed a trend toward significance (*P* = 0.063; Table 2). No treatment × day interaction (*P* = 0.694) was detected for AST (Table 2). All serum variables, except AST, were higher (*P *< 0.001) in DPR than in CON heifers on day 2 after treatment application. In contrast, these serum variables remained similar (*P *> 0.85) after the deprivation event (Figures 3, 4, 5, 6, and 7).

## Discussion

In beef cattle operations, routine management practices such as weaning (Haley et al., 2005) and transportation can lead to water and feed deprivation (Marques et al., 2012, 2019). These events activate the hypothalamic-pituitary-adrenal axis and stimulate the release of cortisol into circulation (Marques et al., 2012, 2019), which stimulates several reactions detrimental to animal performance (Araujo et al., 2010; Cooke, 2017). In addition, the ruminal ecosystem may be compromised, leading to bacterial death (Meiske et al., 1958), which releases endotoxins that might also stimulate an inflammation reaction (Carroll et al., 2009). Consequently, an increase in the plasma concentration of acute-phase proteins such as haptoglobin and ceruloplasmin can be observed (Marques et al., 2012). Although we did not analyze the concentrations of these proteins, we assessed the concentration of the negative acute-phase protein albumin, which is known to exhibit reduced serum concentrations under stressful conditions (Ceciliani et al., 2012). Increased albumin can also be a dehydration marker in livestock animals (Peñuela et al., 2011; Kessell, 2015). In the present study, albumin concentration was elevated after treatment application in deprived animals on day 2, indicating possible dehydration in animals subjected to 48 h of water and feed restriction.

As reported, water and feed deprivation can activate the hypothalamic-pituitary-adrenal axis and stimulate the release of cortisol into the bloodstream (Marques et al., 2012, 2019). Additionally, a lack of food and water promotes the breakdown of muscle and adipose tissue into glycerol and amino acids, respectively, which are needed by the liver to manufacture enzymes and gluconeogenesis (Baxter and Forsham, 1972). In our investigation, 48 h of water and feed restriction did indeed raise serum levels of total protein and NEFA. These results align with earlier research by Marques et al. (2012) and Cole et al. (1986), which discovered that animals deprived of water and food for 24 and 46 h had higher plasma NEFA concentrations and serum total proteins, respectively. In our study, the increase in NEFA concentration indicates that body fat was mobilized while the animals were under nutritional stress. With the increase in NEFA and total protein, the elevated serum glucose concentration at the end of the fast period also suggests that the tissue mobilization stimulated gluconeogenesis to raise the serum glucose concentration and supply energy for essential bodily processes during the deprivation.

Our study observed increased plasma urea concentration in the animals subjected to water and feed deprivation. This increase may be related to the amino acid deamination process involved in glucose production (Baxter and Forsham, 1972). Moreover, blood urea concentration can be enhanced due to AST activity, which might supply amino groups to synthesize urea in the liver from glutamate (Nelson and Cox, 1999). Although AST activity was similar between treatments, this suggests that the increase in urea may have been caused by the deamination of amino acids required in gluconeogenesis rather than by increased AST activity in animals that had been restricted for 48 h.

During water and feed deprivation in commercial farms, animals commonly experience BW loss (Marques et al., 2012, 2019; Beenken et al., 2021), which may be partly due to fecal and urinary losses during these events (Cole et al., 1986). Phillips et al. (1991) reported a 6.8% reduction in BW after 48 h of water and feed deprivation, with 60.9% of the BW loss attributed to fecal and urinary excretion, indicating that other mechanisms may be involved. BW loss during feed and water deprivation in pens or transportation for 8 to 24 h can range from 5.7% to 9.6% (Marques et al., 2012; Deters and Hansen, 2020; Beenken et al., 2021). In our study, the animals were subjected to a prolonged period of water and feed deprivation, with pasture being their primary food source, resulting in a BW loss of 14.6% (246 vs. 210 kg), higher than the values observed in the abovementioned studies.

Over the following 10 d (days 2 to 12), the animals exhibited partial compensatory gain, reducing the difference in BW between treatments to 4.9% (256 vs. 244 kg), with DPR gaining 3.43 kg/d compared to CON gaining 0.996 kg/d. Despite this partial recovery, DPR heifers were still 12 kg lighter than CON by the end of the evaluation period (311 vs. 299 kg). During the rehydration and refeeding period, it is possible to recover BW, but the duration of this process can vary significantly, from 2 to 16 d (Lofgreen et al., 1975; Marques et al., 2019). Our study observed that grazing animals deprived of water and feed for 48 h performed better throughout the experimental period after deprivation. However, this recovery was insufficient to equalize the weight of the animals that had regular access to water and feed throughout the experimental period, resulting in a 12 kg difference at the end of the experiment. This partial compensatory gain may be attributed to the fact that the animals lost not only digestion content, feces, and urine but also body reserves (Ellenberger et al., 1989; Cooke et al., 2007; Marques et al., 2012). In addition, the animals must restore their normal rumen functioning and rumen bacteria throughout the refeeding phase for their feeding consumption to return to normal (Meiske et al., 1958; Galyean et al., 1981). Galyean et al. (1981) found that after feed and water deprivation events, the ruminal microbial community takes 72 h to restore to its starting levels. Cole et al. (1986) and Cole and Hutcheson (1985) found that this disruption in the microbial population can reduce feed intake and ruminal fermentative activity. Prolonged water withdrawal has been found to have a detrimental effect on intake and BW by reducing the size of cattle meals, apparently in an attempt to maintain rumen homeostasis during extended water withdrawal (Utley et al., 1970; Senn et al., 1996; Steiger Burgos et al., 2001). Hence, these processes might explain why animals deprived for 48 h and then returned to pasture did not fully recover their BW throughout the experimental period.

In conclusion, the findings of this study suggest that Nellore (*B. indicus*) heifers subjected to a 48-h deprivation period of water and feed experienced significant alterations in their blood metabolites and lost 14.6% of their BW by the end of the deprivation period. Although the deprived heifers exhibited partial compensatory gain afterward, they remained 12 kg lighter than the non-deprived heifers throughout the production cycle. Nevertheless, further investigation is required to clarify the mechanisms regulating feed intake, performance, hormonal responses, and ruminal fermentation parameters in grazing cattle following a nutrient restriction event.

## Abbreviations

ADF: acid detergent fiber
AST: aspartate aminotransferase
ADG: average daily gain
BW: body weight
CP: crude protein
DMI: dry matter intake
NDF: neutral detergent fiber
NEFA: non-esterified fatty acids
